# A pilot case-control study using a one health approach to evaluate behavioral, environmental, and occupational risk factors for chronic kidney disease of unknown etiology in Sri Lanka

**DOI:** 10.1186/s42522-020-00034-3

**Published:** 2021-02-23

**Authors:** Jake M Pry, Wendi Jackson, Ruwini Rupasinghe, Guneratne Lishanthe, Zied Badurdeen, Tilak Abeysekara, Rohana Chandrajith, Woutrina Smith, Saumya Wickramasinghe

**Affiliations:** 1 Implementation Science Unit, Centre for Infectious Disease Research Zambia (CIDRZ), 10101 Lusaka, Zambia; 2 School of Medicine, Washington University, St. Louis, MO USA; 3 School of Veterinary Medicine, University of California, Davis, USA; 4 Renal Research Centre, District Hospital, Girandurukotte, Sri Lanka; 5 Center for Research and Training on Kidney Diseases (CERTKiD), Faculty of Medicine, University of Peradeniya, Kandy, Sri Lanka; 6 Faculty of Science, University of Peradeniya, Kandy, Sri Lanka; 7 Faculty of Veterinary Medicine and Animal Science, University of Peradeniya, Kandy, Sri Lanka

## Abstract

**Background:**

Chronic kidney disease of unknown etiology (CKDu) was first recognized in Sri Lanka in the early 1990s, and since then it has reached epidemic levels in the North Central Province of the country. The prevalence of CKDu is reportedly highest among communities that engage in chena and paddy farming, which is most often practiced in the dry zone including the North Central and East Central Provinces of Sri Lanka. Previous studies have suggested varied hypotheses for the etiology of CKDu; however, there is not yet a consensus on the primary risk factors, possibly due to disparate study designs, sample populations, and methodologies.

**Methods:**

The goal of this pilot case-control study was to evaluate the relationships between key demographic, cultural, and occupational variables as risk factors for CKDu, with a primary interest in pesticide exposure both occupationally and through its potential use as an ingredient in brewed kasippu alcohol. An extensive one health focused survey was developed with in cooperation with the Centre for Research, Education, and Training on Kidney Diseases of Sri Lanka.

**Results:**

A total of 56 CKDu cases and 54 control individuals were surveyed using a proctored, self-reported questionnaire. Occupational pesticide exposure and alcohol consumption were not found to be significant risk factors for CKDu. However, a statistically significant association with CKDu was observed with chewing betel (adjusted odds ratio [aOR]: 6.11, 95% confidence interval [CI]: 1.93, 19.35), age (aOR: 1.07, 95% CI: 1.02, 1.13), owning a pet dog (aOR: 3.74, 95% CI: 1.38, 10.11), water treatment (aOR: 3.68, 95% CI: 1.09, 12.43) and pests in the house (aOR: 5.81, 95% CI: 1.56, 21.60).

**Conclusions:**

The findings of this study suggest future research should focus on practices associated with chewing betel, potential animal interactions including pests in the home and pets, and risk factors associated with water.

**Supplementary Information:**

The online version contains supplementary material available at 10.1186/s42522-020-00034-3.

## Introduction

There has been a notable increase in the recognized incidence of chronic kidney disease (CKD) around the world [1]. Kidney disease has moved from 27th most common cause of death in 1990, to 18th in 2010 and has come to be considered a global public health problem causing high morbidity, mortality, and financial burden [2–4]. Global prevalence of CKD is estimated to range between 8 and 16%, and differs substantially across developed and developing countries [3, 5]. Although diabetes mellitus and hypertension remain the leading causes of CKD, in recent years a different form of CKD has reached epidemic levels, creating serious public health complications in rural communities in the dry zone of Sri Lanka [6, 7]. The recognition of endemic CKD in the dry zone in the 1990s coincided with the development of the rural healthcare system, which improved access to clinics by affected individuals. Since that time, the dry zone has seen a disproportionate increase in cases of CKD compared to the rest of the country [8]. Existing studies describe the majority of these CKD patients as not having hypertension or diabetes mellitus, two of the major risk factors for CKD. It has therefore, been defined as a distinct condition: CKD of unknown or uncertain etiology (CKDu). Similar chronic kidney disease hotspots have been recognized among farmers in Central America (Nicaragua and El Salvador) and South Asia [9–11].

Approximately 2.5 million people live in the subset of Sri Lankan provinces where CKDu is most common [12, 13]. Cases of this disease predominate in the Medawachchiya, Wilgamuwa, Nikawewa, and Girandurukotte regions of the dry zone. Studies have shown the highest prevalence of CKDu among 30–60 year old men engaged in chena or rice farming, and estimate a total of 20,000 (approximately 0.8% population) affected in the North Central Province [8, 14].

The epidemic of CKDu in the dry zone is burdening the rural healthcare system and impacting agricultural productivity due to a reduction in the available labor force when CKDu patients are too ill to work [15–17]. Due to the irreversible and progressive nature of CKD, most patients require long-term dialysis since renal transplants are not commonly available. For these reasons, there is a need to determine the risk factors associated with CKDu to control and attenuate the incidence of new CKDu cases. Previous study regarding the relationship between alcohol consumption and increased transdermal pesticide penetration served to motivate examination of the relationship between alcohol consumption and pesticides and CKDu diagnosis [18, 19]. Further to this, pesticides were considered a alone as a risk for CKDu given established nephrotoxicity [20]. A growing body of evidence suggests that CKDu is multi-factorial, making it difficult to identify individual risk factors and potential interactions involved in pathogenesis [7, 13, 21–24]. Recently, various heavy metal agents such as cadmium, arsenic agrochemicals, aluminum, and fluoride, as well as infectious diseases such as leptospirosis have been considered for association with CKDu [25–30].

Collaboration between researchers at the University of Peradeniya in Sri Lanka, the University of California, Davis (UCD) in the United States, and Sri Lankan stakeholders in CKDu-endemic areas were involved in this pilot study. The driving hypothesis for this study is that alcohol consumption and/or pesticide exposure are associated with CKDu as a health outcome. In addition, it is recognized that relationships between key demographic, cultural, and occupational variables may play a role in CKDu health outcomes [18, 19, 31–33].

## Methods

A pilot case-control study was conducted in Sri Lanka from July–October 2015. This time interval coincides with the low farming activity season and availability of local research team. The study population was comprised of individuals (cases and controls) who resided in the North Central Province (NCP) or Uva Province (UP), participants in community CKDu testing outreach, and/or sought medical care at Girandurukotte district hospital (UP) or Medawachchiya clinic (NCP). The population in both the NCP and UP is approximately 1.2 million, with women making up the slight majority (51%) [14]. The majority of people in both provinces are Sinhalese-speaking and resides in the rural sector where they engage in farming (chena, rice). The NCP has the highest recorded prevalence of CKDu cases in Sri Lanka and is located in the country’s dry zone. Uva Province is in the intermediate zone adjacent to the dry zone, with a lower prevalence of CKDu cases compared to the NCP. Recruiting cases and controls from the same community outreach effort helped reduce potential bias associated with health-seeking behavior.

We tested the hypothesis that there is a relationship between alcohol consumption and CKDu diagnosis, and pesticide exposure and CKDu, in endemic areas of Sri Lanka, a questionnaire survey was developed [See Additional file 1]. The survey tool encompassed a wide range of exposures to capture potential unknown confounders, including exposures suggested by local CKDu working groups at the University of Peradeniya. Individuals meeting the CKDu case definitions as well as a comparison (control) population from the same endemic region were invited for participation in the survey.

Due to constraints associated with the sample of those that responded to the invitation for a follow-up interview cases and controls were not matched. Given that this was a pilot study we allowed for identifications of associations across all covariates. Insight from local experts provided confidence that there was sufficient heterogeneity across our wide scope of covariates.

### Case definition

The most common method for CKDu diagnosis in Sri Lanka involves examination of albumin-creatinine ratio and/or persistent proteinuria. Individuals diagnosed with definite or probable CKDu by a nephrologist at the Girandurukotte regional hospital or Medawachchiya renal clinic made up the population from which study cases were selected. An individual was considered a definite CKDu case if creatinine levels were elevated and subsequent renal biopsy the finding was predominant tubular interstitial nephritis. A probable CKDu case was defined as persistent renal dysfunction for more than 3 months after excluding known causes including hypertension, diabetes mellitus, any other known renal diseases. The control group was defined as those for which negative results for CKDu from hospital or population screening records were available/confirmed and no current CKDu diagnosis was self-reported.

### Recruitment

All participants were recruited through CKDu screening efforts of the Girandurukotte regional hospital or Medawachchiya renal clinic (study facilities). Individuals were eligible to participate as a control if they had previously screened CKDu negative at one of the community CKDu screening initiatives supported/sponsored by the two study facilities in the past 3 years as confirmed by clinical staff review and did not, upon return, report having been diagnosed with CKDu. Potential control participants were invited via post (hard copy letter) to return to the Girandurukotte regional hospital or Medawachchiya renal clinic to take part in our survey. Approximately 100 invitations were sent to potential control participants across the two study facilities. All cases were recruited from Girandurukotte regional hospital or Medawachchiya renal clinic and had been in care for CKDu for less than 5 years. Invitation for survey participation among cases was directed by clinical partners who selected those most recently diagnosed with CKDu at one of the study facilities until target enrollment was attained. All participation in the survey was completely voluntary.

### Sample size calculation

The total sample size calculated for this pilot case-control study was 110, comprising 1:1 cases to controls. The target sample size of 110 individuals was calculated based on a power of 80% (β = 0.2), 95% confidence (α = 0.05), and a minimum effect size of 3.0. This relatively large effect size was considered in the exploratory study in order to identify preliminary exposures strongly associated with the outcome. An estimate of 26% was used for any reported alcohol consumption among controls for the sample size calculation [34, 35].

### Survey design

Survey questions were designed by the research team in consultation with the resource personnel at the Centre for Research, Education, and Training on Kidney Diseases (CERTkID) in Sri Lanka prior to IRB approval, survey training, and interviews. The survey consisted of 138 questions structured as binary, categorical, ordinal, and open-ended across six categories: 1) agricultural information; 2) animal exposure; 3) water and nutrition; 4) alcohol consumption; 5) respondent demographics; and 6) family and past medical history.

The agricultural information section included questions related to farming practices and agrochemical usage. Information on ownership and health of livestock and pets, presence of pest animals and wildlife were collected in section two of the survey. In the water and nutrition information section, sources for drinking, cooking, and bathing water were assessed, along with participant practices regarding water treatment prior to use. The alcohol consumption information section contained questions on type of alcohol consumed, betel chewing and smoking status. Alcohol consumption was assessed in two ways: a binary question was asked first on whether the participant had ever consumed alcohol (if yes, what type and frequency) and second, whether the participant believed that alcohol was a problem in their village. The respondent demographics section contained questions pertaining to level of education and family income. To assess a potential genetic component of CKDu, participants were asked whether their spouses were close blood relatives and family history of CKDu, hypertension, and diabetes mellitus was also recorded. Detailed survey and explanation of survey components are given in Supplementary information.

Survey data were collected by 12 trained graduate students associated with the University of Peradeniya. In addition, investigators were present at each site during data collection, allowing surveyors the opportunity for clarification as needed in real-time as interviews were conducted. Cases and controls participated voluntarily, and surveys were administered verbally in the mother tongue of the participants (Sinhala) after obtaining consent (see Supplementary Information for the English version of the consent form and survey questions). Survey question responses were recorded on paper copies of the questionnaire by the surveyor. Each interview took approximately 1 h to complete. All survey responses were data entered, from hard copy into a secure database by research coordinators.

The research protocol was designed according to the guidelines of the International Compilation of Human Research Standards (2015 edition) and approved by the University of California, Davis Institutional Review Board (#762486–2). Written consent of all study participants was obtained by signature or thumbprint after survey enumerators verbally read the consent statement in the appropriate language. The consent form was translated in both Sinhala and Tamil. The majority of survey interviews took place at medical clinics specializing in renal disease.

### Statistical analyses

Logistic regression was used to evaluate risk factors for CKDu case status. Potential risk factor association with the outcome of interest was assessed and a cutoff *p*-value ≤0.20 was used to restrict consideration for the final model. Pearson’s correlation coefficient was used to identify covariate correlation at − 0.5 ≤ *ρ* ≥ 0.5. Data analysis was completed using Stata IC 14 (StataCorp LP, College Station, TX USA). Multiple logistic regression analyses were performed using backwards stepwise selection to model the risk factors associated with the CKDu disease outcome of interest. Adjusted analysis was done to control for possible confounding by measured covariates. Statistical significance was assessed at the *α* = 0.05. Mapping was completed using QGIS v2.18 (Free Software Foundation, Inc. Boston, MA 02110–1301 USA) with GADM administrative boundary layers v2.8 (accessed November 2015).

## Results

A total of 110 participants were included in the analysis; 56 met the case definition and 54 satisfied control criteria (Table S1). All participants resided in the CKDu endemic regions in Girandurukotte and Medawachchiya districts in Sri Lanka at the time of diagnosis. Participants had a mean age of 52.6 years (range = 25–80); there was a slight majority of males (60%) to females (40%). Most participants reported to be married, with about half reporting being married to a spouse that was a close blood relative and slightly over half reporting a family member having been diagnosed with CKDu (Table S1). Of the 110 study participants, half reported consuming any type of alcohol and the majority reported using some type of pesticide in their daily lives (insecticide, herbicide, in-home pesticide and/or fungicide) (Table S1).

**Fig. 1:**
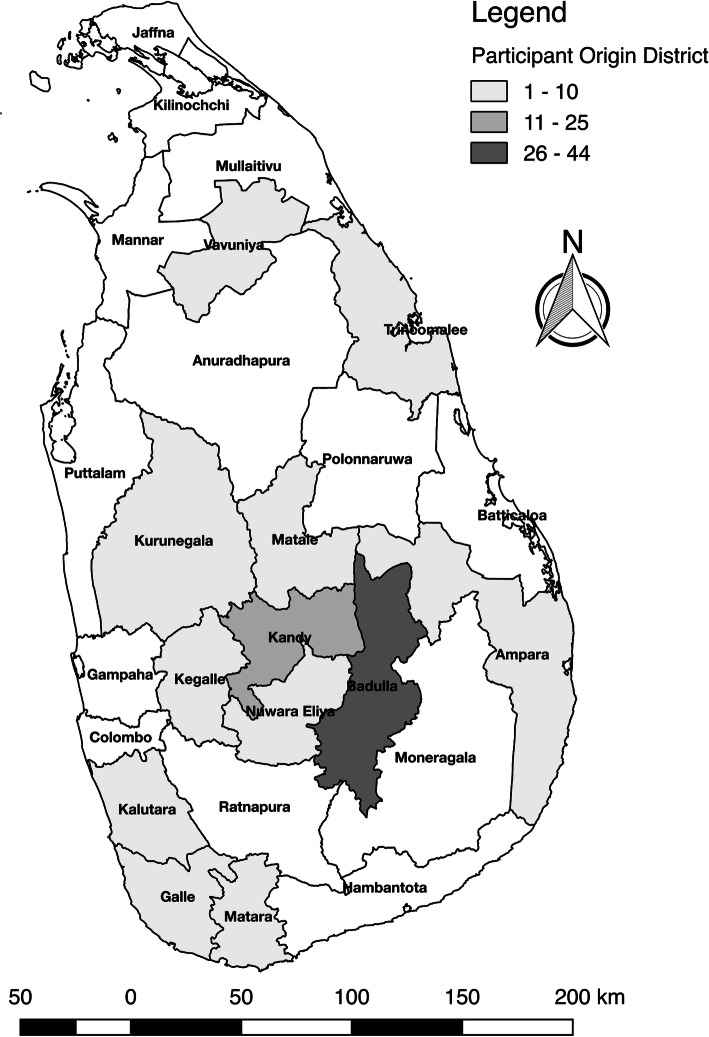
Map of Sri Lanka by district indicating participant’s birth district

The majority of participants (74) were residing in the Uva Province. Twenty-two participants resided in the North Central Province, five in the Eastern Province, two in the Central Province and one resided in the Northwestern Province. There were 8 (7.3%) individuals for whom reliable current residence information was not available due to survey legibility and standardization complications. Data regarding province in which the participant was born were collected (Fig. 1). No participants reported birth outside of Sri Lanka.

After bivariate and correlational analyses, smoking unfiltered cigarettes and smoking cannabis were found to be highly correlated (correlation coefficient = 0.5141 > 0.5 threshold). Smoking unfiltered cigarettes was dropped from model consideration given a bivariate *p*-value higher than that of smoking cannabis (*p-*value = 0.186 and 0.112, respectively).

Sources of drinking water were surveyed and ‘dug well’ was the most common source (90.9%) of drinking/cooking water, with ‘rainwater collection containers’ being the second most common source. Drinking water was reported to be treated with routine methods such as boiling or filtering, or traditional methods such as placing igini (*strychnos potatorum*) seeds in the water source/well [36]. A subset of key population characteristics is reported in Table 1.

**Table 1:** Study Population Characteristics by Case-Control Status

*Factor*	*Level*	*Control* *n (%)*	*Case* *n (%)*	*P-Value*
*N*		*54*	*56*	
Age, mean (SD)*		49.5 (11.7)	57.5 (9.6)	< 0.001
Gender*	Female	27 (51)	16 (29)	0.017
	Male	26 (49)	40 (71)	
Diabetes mellitus Diagnosis	Yes	13 (24)	2 (4)	< 0.001
	No	41 (76)	54 (96)	
Farming as occupation*	No	14 (28)	3 (6)	0.002
	Yes	36 (72)	51 (94)	
Drinking water source	Dug Well	48 (89)	52 (93)	0.47
	Rain Water	6 (11)	5 (9)	0.7
Treat drinking water	Drinking	34 (63)	41 (75)	0.19
Keep livestock	Livestock	17 (31)	19 (34)	0.78
Smoking status	Tobacco	8 (15)	14 (25)	0.18
	Cannabis	2 (4)	7 (12)	0.092
Chew betel*	Betel	22 (41)	40 (71)	0.001
Alcohol a problem in village	Not a Problem	13 (27)	11 (21)	0.85
	Minor Problem	17 (35)	20 (38)	
	Moderate Problem	10 (20)	9 (17)	
	Major Problem	9 (18)	12 (23)	
Alcohol consumption	Any Alcohol	22 (41)	33 (59)	0.056
	Arrack	19 (35)	27 (48)	0.17
	Beer	14 (26)	16 (29)	0.76
	Kasippu	13 (24)	18 (32)	0.35
Pesticide use*	Any	42 (78)	52 (93)	0.025
	Fungicide	21 (44)	29 (55)	0.27
	Herbicide	41 (76)	51 (91)	0.032
	Insecticide	42 (78)	44 (79)	0.92

*Significance attributed as *p*-value < 0.05

There is a significant difference between age, gender, occupation, chewing betel, pesticide use at the 95% confidence level. The cases were older, on average, by 8 years than the control group with a gender imbalance finding males more often among the cases compared to controls. Cases cited farming as an occupation more often than controls (22% higher among cases), reported chewing betel (30% higher among cases), and used pesticides more often, specifically herbicide, (15% higher among cases).

Two multivariable models were constructed, an exposure of interest model and an exploratory model. The exposure of interest model forced inclusion of variables concerning alcohol consumption and pesticide exposure, as neither exposure of interest was found to be significantly associated with CKDu in bivariate analysis (Table 2). The final exposure of interest model included four variables and excluded one variable compared to the stepwise method used for the exploratory model (Table 3). The exploratory model (Table 3) included only risk factors significantly associated with CKDu status (*P* < 0.05). The primary exposures of interest (alcohol consumption and pesticide exposure) were not found to be significant using a backward stepwise selection process. However, age – considered as a continuous variable (aOR: 1.08, 95% CI: 1.02, 1.13), chewing betel (aOR 4.01, 95% CI: 1.49, 10.81), keeping a pet dog (aOR: 4.21, 95% CI: 1.55, 11.48), and reporting pests in the home (aOR: 3.96, 95% CI: 1.21, 12.93) were significantly associated with CKDu case status.

**Table 2:** Adjusted Odds Ratios for CKDu Using Backward Stepwise Approach with Forced Inclusion of Primary Exposures of Interest – Pesticide Use and Alcohol Consumption

Factor	Odds Ratio	***P***-Value	95% Confidence Interval
Age*	1.12	< 0.01	(1.04, 1.21)
Sex: male	6.19	0.07	(0.73, 44.06)
Chew betel	3.57	0.08	(0.86, 14.84)
Pet dog*	4.41	0.03	(1.19, 16.27)
Pests in-home*	8.19	0.02	(1.45, 46.18)
Consume arrack	0.64	0.62	(0.11, 3.86)
Consume beer	1.15	0.88	(0.18, 7.34)
Consume kasippu	0.26	0.23	(0.03, 2.39)
Fungicide	1.45	0.57	(0.41, 5.17)
Herbicide	1.00	1.00	–
In-home pesticide	0.82	0.79	(0.34, 4.97)
Insecticide	1.00	1.00	–

Note: Exposures of interest kept in model despite non-significant *p*-value

*Significance attributed as *p*-value < 0.05

**Table 3:** Adjusted Odds Ratios for CKDu Using a Backward Stepwise Approach

Factor	Odds Ratio	***P-***Value	95% Confidence Interval
Chew betel*	5.95	0.002	(1.88, 18.86)
Pet dog*	3.515	0.012	(1.31, 9.42)
Treat water*	3.944	0.026	(1.18, 13.24)
Pests in-home*	5.708	0.009	(1.54, 21.18)
Age*	1.078	0.003	(1.03, 1.13)

*Significance attributed as *p*-value < 0.05

Bivariate and adjusted analyses for specific types of pesticides and fertilizers used are detailed in Table 4. Before adjustment for age, gender, occupation, and alcohol consumption, usage of a fertilizer (muriate of potash) and an herbicide (glyphosate) were significantly associated with CKDu diagnosis. In an adjusted analysis, we found no significant associations among fertilizers, insecticides, or herbicides reported with diagnosis of CKDu (Table 4).

**Table 4:** Crude and Adjusted Odds Ratios for Agrochemical Association with CKDu

Factor	Control (***N*** = 54) n (%)	Case (***N*** = 56) n (%)	Crude Odds Ratio (95% CI)	Adjusted Odds Ratio (95% CI)
**Fertilizer**				
Urea	38 (70%)	47 (84%)	2.20 (0.9, 5.5)	0.92 (0.3, 3.2)
Muriate of Potash*	1 (2%)	0 (0%)	3.15 (1.4, 7.4)	1.86 (0.7, 5.1)
Triple Super Phosphate	11 (20%)	21 (38%)	2.35 (1.0, 5.5)	1.84 (0.7, 5.2)
Mud/Manure	11 (20%)	5 (9%)	0.38 (0.1, 1.2)	0.41 (0.1, 1.5)
**Insecticide**				
Carbosulfan	5 (9%)	3 (5%)	0.55 (0.13, 2.44)	0.48 (0.1, 2.5)
Carbofuran	4 (7%)	4 (7%)	0.96 (0.23, 4.06)	0.47 (0.1, 2.4)
Curateer	7 (13%)	6 (11%)	0.81 (0.25, 2.57)	0.74 (0.2, 2.8)
**Herbicide**				
Glyphosate*	19 (35%)	32 (57%)	2.46 (1.14, 5.30)	1.09 (0.4, 2.8)
MCPA	24 (44%)	28 (50%)	1.25 (0.59, 2.65)	0.92 (0.4, 2.3)
DPA	11 (20%)	17 (30%)	1.70 (0.71, 4.08)	0.88 (0.3, 2.5)
Metamifop	8 (15%)	8 (14%)	0.96 (0.33, 2.77)	1.39 (0.4, 4.9)

* *p* ≤ 0.05; Adjusted by age, gender, farming occupation and alcohol consumption

**Fig. 2:**
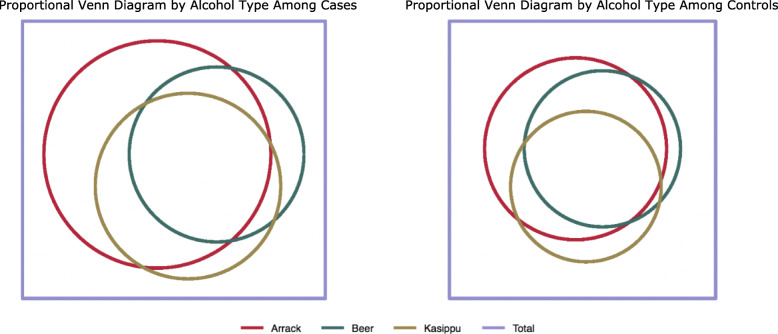
Proportional Venn Diagrams Representing Reported Alcohol Type Consumed by Case-Control Status

Figure 2 illustrates the difference between cases (A) and control (B) by type of alcohol reportedly consumed. Cases were more likely to report drinking. The overlap between all three types of alcohol indicates that if one consumes alcohol it is common to drink all three types surveyed. Arrack was the most commonly reported alcohol consumed across cases and controls. Overall reported alcohol consumption was observed among 2.3% of women, while 81.8% of men reported drinking alcohol (two-sided Fisher’s exact: < 0.001).

## Discussion

There was no significant association detected between CKDu and pesticide exposure nor alcohol consumption. However, there were significant associations identified for chewing betel, owning a pet dog, treatment of drinking water, reporting pests in the home, and age. These significant exposures provide insight into previously unconsidered routes and mechanism for CKDu in addition to potential guidance on how to reduce odds of CKDu diagnosis. Chewing betel could be a risk directly or indirectly through contamination of the traditional chew ingredients or through handling and/or preparation of the betel chew. The association between report of a pet dog could suggest a zoonotic pathway and pests in the home could indicate pest extermination agent risks or a disease reservoir. Treatment of drinking water, especially boiling, may contribute to consumption of aluminum with nephrotoxic consequences. The wide variety of associated exposures suggests that there may be multiple risk factors associated with CKDu, which is consistent with results of previous studies [37, 38].

It is important to note that those cases reporting ever having been diagnosed with diabetes or hypertension were diagnosed after meeting the CKDu case definition. Prevalence of diabetes among controls was high (24.1%) relative to the 2011 and 2030 national estimates of 7.8 and 9.1%, respectively [39]. The questionnaire did not differentiate between type I or type II diabetes mellitus, limiting inferences about reasons for this discrepancy in prevalence. It is possible that changes in meal preparation (OR = 1.60 95% CI: 0.74–3.44) and/or food stuffs available in rural Sri Lanka also play a role in the increased prevalence of diabetes in this control sample. Conversely, diagnosis of hypertension is significantly higher (Fisher’s exact = 0.002) among the cases. However, this is common sequelae of chronic kidney disease [40–42].

Uses of a variety of agrochemicals are common throughout the farming dry zone regions and are often readily available through government subsidies. In our study, only herbicide use was shown to be significant in the bivariate analysis among all insecticide, fungicide, and in-home pesticide parameters. It is possible that exposure to pesticide occurs among those farmers reporting no use of pesticides on their crops through adjacent farm pesticide use in tandem with dynamic environmental factors, i.e. flooding, water source contamination, and winds.

The ingredients used in making kasippu, an illicit locally brewed alcohol, were of special interest in this study, due to prior hypothesis that pesticides are introduced in the brewing process. However, study participants who reported drinking kasippu most often purchased it from other villagers and either did not know the ingredients used or did not want to report drinking kasippu due to it being an illicit form of alcohol in Sri Lanka as well as the perceived cultural stigma for reporting use. Of those that did report drinking kasippu, some reported urea as an ingredient in kasippu production, which could have potentially toxic biologic effects, leading to increased blood urea nitrogen subsequently impacting kidney function [43, 44].

Previous studies have suggested that drinking water quality and contamination may be associated with CKDu [45–47]. Prior studies have identified cases and controls on the basis of groundwater source and found much larger odds of disease in males who drank water from shallow wells, compared to males who drank from natural springs (OR 5.48 95% CI: 3.46–8.66) [48, 49]. Similar findings were found for women drinking from shallow wells (OR 4.40 95% CI: 2.23–8.68) [48]. Due to the broad application of pesticides in all aspects of farming, potentially nephrotoxic pesticide agents contaminating the drinking water cannot be ruled out. Our findings differ somewhat from these prior studies in the minor difference between 89% (controls) and 93% (cases) reported source of drinking water as a dug well. We however, were not able to compare other sources of drinking water with high confidence and statistical power.

Dug wells are traditional wells often lined with clay brick and may be covered to prevent animals from entering. There were few participants who received water via a tap line, rainwater collectors, or methods other than a dug well. As such, our ability to evaluate drinking water source as a risk factor was limited. In addition, the number of years that participants used different types of drinking water was inconsistently recorded. Treatment of drinking water was found to be a significant risk factor for CKDu. Treatment of water included boiling water (*n* = 41), filtering water (*n* = 59), and traditional methods (*n* = 19). The most common traditional practice for water treatment was the introduction of *Strychnos potatorum* seeds (Sinhalese - ingini seeds) into the water source, as is customary in Sri Lanka and India [50]. One possible risk for developing CKDu related to treating drinking water could be the boiling (*n* = 41) of water in aluminum vessels [51]. Information regarding the type of cookware used with relation to boiling water was not collected.

Results regarding the potential mechanisms of association with CKDu for chewing betel, treatment of drinking water, and having pets or pests are inconclusive. However, it is possible that ownership of dogs in Sri Lanka, often community pets, may be difficult to ascertain, although 43% of study participants reported having a pet dog. These community dogs may serve as reservoirs of infectious disease such as leptospirosis. Outbreaks of leptospirosis have been reported during the monsoon season in some CKDu endemic regions [52, 53]. Consequently, dogs may potentially shed *Leptospira* spirochetes in their urine leading to community exposure. Humans infected with *Leptospira* through canine urine might passively clear the infection and experience renal damage that could later lead/contribute to the developments of CKDu [54]. Current Ministry of Health CKDu diagnosis criteria do not include serology or polymerase chain reaction (PCR) tests for ruling out leptospirosis; patients are asked only to self-report previous history of leptospirosis. As leptospirosis diagnostics continue to improve, future research should consider specimen collection for laboratory confirmation [55–57].

Mammalian pests in households may also be carriers of infectious disease that leads to increased susceptibility to CKDu. There is an emerging hypothesis that Hanta virus could be the possible causative agent for CKDu in Sri Lanka’s dry zone [30, 58]. Humans infected with Hanta virus show clinical signs similar to those of Leptospirosis, and Hanta virus infection in humans was first described in Sri Lanka in the mid-eighties [59]. Rodents act as the reservoir host for Hanta virus and *Leptospira*. Our study, therefore, may provide further evidence for the mechanism of increased CKDu prevalence through rodent pests and pet(s) serving as a potential route/intermediate for Hanta virus and/or *Leptospira* in humans in Sri Lanka. Further research should evaluate the prevalence of Hanta virus and *Leptospira* in rodents, pets, livestock, and people in CKDu-endemic regions in Sri Lanka. Additionally, *Leptospira* vaccination campaigns may be considered a potential intervention model to address CKDu.

To identify other disease risks that might be due to close contact with domestic animals or wildlife, data was collected concerning participant observations of illness among domestic animals in the community. There were very few reported, including three observations of ill cattle (2.7%), one goat (0.9%), and one chicken (0.9%) in the month prior to the survey interview. It is possible that the lifespan of dogs and livestock in the community is short due to environmental hazards (disease, trauma, etc.) and animals go missing or die before disease can be detected or transmitted to humans.

Chewing betel was another novel risk factor for CKDu in our study. This practice is quite common among Sri Lankans, and our study found that chewing betel was more common among those reporting farming as an occupation (60%) than other occupations (40%). There is evidence that betel preparations include stimulant properties similar to nicotine, and chewing it routinely can lead to enamel erosion and oral cancer [60–62]. The betel preparation commonly chewed in Sri Lanka is comprised of betel, areca nut, tobacco, and lime. However, betel recipes among farmers in Sri Lanka’s dry zone may contain differing substances compared to preparations in the remainder of the country, which could be associated with CKDu [60, 63, 64]. Individuals that mix and distribute pesticides may be at greater risk, as betel is inserted in the mouth and may be done in the field where hand washing is not possible. In addition, there is evidence that chewing betel increases exposure to arsenic and cadmium, both of which can be nephrotoxic [65].

Additionally, at our sample size (*n* = 110), we do not have the power to detect small differences in effect measure or to generalize to populations outside our study region. Finally, although survey questions were detailed in nature, the question interpretation by the study participant may have varied, leading to inaccurate answers. Due to the condensed nature of the survey timeline, with multiple interviews occurring at one time, investigators could not oversee each individual interview. As such, details pertaining to the number of years when specific pesticides or water sources were used were sometimes incomplete.

This case-control study design was useful for having comparable populations with and without the disease in order to efficiently evaluate past risk factors associated with disease status. In the future, a cohort study would be a useful design to evaluate exposure data, exposure timelines, and incidence rates for CKDu, and we recommend that future studies of CKDu in Sri Lanka be cohort-based, despite the longer follow-up period and greater expense. In addition, a nationwide, coordinated CKDu research consortium spanning all major research institutions would make CKDu research more efficient by standardizing study design and methodologies. This would allow more accurate conclusions to be drawn from studies with clear and consistent case/control definitions and study locations.

While our survey was comprehensive, our study had several limitations, primarily relating to case definition, disease progression, and study design characteristics. Temporal bias might have been introduced since the exact date of CKDu diagnosis was not collected however, the impact of bias on alcohol consumption is believed to be minimal due to a non-significant difference in the mean years since first drink (t = − 1.89) and frequency of alcohol consumption (t = − 0.49). Furthermore, there was not a significant difference in the number of years farming among those reporting a farming occupation (Fisher’s exact = 0.07) between cases and control.

The appearance and progression of CKDu can involve non-specific symptoms, making the disease challenging to diagnose in the early, pre-clinical stages limiting the population of cases in this study to those that were in advanced stages of the disease. This may have caused the inclusion of false-negatives, sub-clinical cases, in our control group however, without further testing the extent of this issue is unknown. It is possible that survivor bias also played a role in the control group selection making the control group more resistant to the outcome however, the given the age of the control group this effect is likely small. Additionally, there is potentially some historical exposures that increase CKDu diagnosis potential given the younger mean age of control participants. Studies suggest using more sensitive methods for detecting early CKDu, with measurement of microalbuminuria and functional markers such as cystatin C, creatinine or tubular proteins like RBP4, NGAL or KIM would be beneficial in capturing a greater number of early-stage CKDu cases so that exposures can be better assessed and treatment initiated earlier [66–68].

The lack of association between herbicide and CKDu outcome in the adjusted model indicates either that herbicide alone is not responsible for CKDu, or that sufficient detail regarding herbicide use was not captured in the survey. For example, the volume and length of use of pesticides was difficult to assess in an interview setting compared to a household visit, where farmers could reference pesticide receipts or other family members for details they could not recall. More detailed responses regarding usage may have resulted in a difference between cases and controls, which could not be evaluated in this study.

At present, the use of the albumin-creatinine ratio or persistent proteinuria as an initial screening tool has limited capacity to detect CKDu cases before advanced-stage(s). This could cause misclassification of the disease outcome if the diagnostic test sensitivity is not high enough to differentiate between early-stage CKDu patients and controls.

## Conclusion

In conclusion, this pilot case-control study showed that chewing betel, reporting of in-home pets and pests, treating drinking water, and age were significantly associated with CKDu. These cultural and environmental factors are likely part of a multi-factorial etiology that is challenging to unravel, and that may take years to understand whether preventive measures are effective. Future studies should be cohort in design and focus on further exploring the identified risk factors and their epidemiologic relationships to CKDu, as well as possible interventions to attenuate the incidence of CKDu in Sri Lanka. Potential interventions to be considered based on these findings might include safe home pest control options, testing and treatment for leptospirosis among community dogs, routine chronic kidney disease screening among those in CKDu endemic areas, and education focused on hand hygiene in the field. These findings should be considered as research regarding CKDu in other endemic regions continues.

## Supplementary Information

**Additional file 1.**

## Data Availability

Full questionnaire made available in Additional file 1.

## Abbreviations

aOR: Adjusted odds ratio
CERTkID: Centre for Research, Education, and Training on Kidney Diseases
CI: Confidence interval
CKDu: Chronic kidney disease of unknown etiology
DPA: 2,2-dichloropropionic acid
MCPA: 2-methyl-4-chlorophenoxyacetic acid
NGAL: Neutrophil gelatinase-associated lipocalin
KIM: Kidney Injury Molecule
OR: Odds ratio
PCR: Polymerase chain reaction
RBP4: Retinol binding protein 4
SD: Standard deviation
