# Supplementary material for: A pilot case-control study using a one health approach to evaluate behavioral, environmental, and occupational risk factors for chronic kidney disease of unknown etiology in Sri Lanka
Source: One Health Outlook. 2021 Feb 23;3:4. doi: 10.1186/s42522-020-00034-3 (PMC8011406; doi:10.1186/s42522-020-00034-3)
Supplement: Supplementary file 1 — Additional file 1. [file 42522_2020_34_MOESM1_ESM.docx]

**Additional File 1**

**Table S1. Characteristics of Population Surveyed**

| **Factor** | **Category** | **Control**  **(N=54)**  **n (%)** | **Case**  **(N=56)**  **n (%)** | **P-Value** |
| --- | --- | --- | --- | --- |
| **Respondent Demographics and Health** | | | | |
| Gender | Male | 26 (49) | 40 (71) | 0.02 |
| Age, mean (SD) |  | 49.5 (11.70) | 57.5 (9.59) | <0.01 |
| Most Villagers Related^‡^ | Yes | 23 (43) | 33 (59) | 0.09 |
| Education Level | No formal schooling | 5 (9) | 9 (16) | 0.04 |
|  | Primary | 14 (26) | 27 (49) |  |
|  | Middle | 15 (28) | 11 (20) |  |
|  | Secondary | 16 (30) | 7 (13) |  |
|  | > Secondary | 3 (6) | 1 (2) |  |
| Income during Maha season  (from Sep-March) | <25,000 LKR | 6 (12) | 12 (22) | 0.14 |
|  | ≥25,000 to <50,000 LKR | 10 (21) | 14 (26) |  |
|  | ≥ 50,000 to <100,000 LKR | 14 (29) | 17 (31) |  |
|  | ≥100,000 LKR | 15 (31) | 6 (11) |  |
|  | Unknown/declined | 3 (6) | 5 (9) |  |
| Income during Yala season  (from May-Aug) | <25,000 LKR | 9 (23) | 17 (40) | 0.08 |
|  | ≥25,000 to <50,000 LKR | 10 (26) | 14 (33) |  |
|  | ≥ 50,000 to <100,000 LKR | 8 (21) | 7 (17) |  |
|  | ≥100,000 LKR | 10 (26) | 2 (5) |  |
|  | Unknown/declined | 2 (5) | 2 (5) |  |
| Medical care provider | Private | 5 (9) | 0 (0) | 0.01 |
|  | Government | 29 (54) | 42 (76) |  |
|  | Other | 20 (37) | 13 (24) |  |
| Annual medical expenses | No Expense Declared | 5 (9) | 6 (11) | 0.06 |
|  | <1,000 LKR | 17 (31) | 6 (11) |  |
|  | ≥ 1,000 to < 10,000 LKR | 22 (41) | 29 (52) |  |
|  | ≥10,000 LKR | 10 (19) | 15 (27) |  |
| Average annual clinic visits | 1-6 times | 22 (43) | 3 (5) | <0.01 |
|  | 7-12 times | 14 (27) | 17 (30) |  |
|  | 13-18 times | 9 (18) | 19 (34) |  |
|  | >18 times | 6 (12) | 17 (30) |  |
| Chew betel | Yes | 22 (41) | 40 (71) | <0.01 |
| Smoke tobacco/cannabis | Yes | 14 (26) | 19 (34) | 0.36 |
| **Agricultural Information** | | | | |
| Occupation farming | Yes | 36 (72) | 51 (94) | <0.01 |
| Use insecticide | Yes | 42 (78) | 44 (79) | 0.92 |
| Use fungicide | Yes | 21 (44) | 29 (55) | 0.27 |
| Use pesticide in home | Yes | 9 (20) | 12 (22) | 0.87 |
| Use herbicide | Yes | 41 (76) | 51 (91) | 0.03 |
| Agro-mechanization | None | 10 (21) | 9 (17) | 0.76 |
|  | Some | 10 (21) | 11 (21) |  |
|  | Much | 11 (23) | 17 (33) |  |
|  | All | 16 (34) | 15 (29) |  |
| Farm type | Highland | 2 (4) | 5 (9) | 0.42 |
|  | Lowland | 41 (85) | 45 (85) |  |
|  | Highland & Lowland | 5 (10) | 3 (6) |  |
| Use pesticide spray PPE | Yes | 14 (56) | 25 (68) | 0.35 |
| **Alcohol Consumption** | | | | |
| Consume kasippu | Yes | 13 (24) | 18 (32) | 0.35 |
| Consume arrack | Yes | 19 (35) | 27 (48) | 0.17 |
| Consume beer | Yes | 14 (26) | 16 (29) | 0.76 |
| Consume other alcohol | Yes | 8 (15) | 12 (21) | 0.37 |
| Perceived alcohol problem in village | No problem | 13 (27) | 11 (21) | 0.85 |
|  | Minor problem | 17 (35) | 20 (38) |  |
|  | Moderate problem | 10 (20) | 9 (17) |  |
|  | Major problem | 9 (18) | 12 (23) |  |
| **Water and Nutrition** | | | | |
| Drink dug well water | Yes | 48 (89) | 52 (93) | 0.47 |
| Drink rainwater | Yes | 6 (11) | 5 (9) | 0.70 |
| Treat drinking water | Yes | 34 (63) | 41 (75) | 0.19 |
| Meal preparation changed | Yes | 19 (35) | 26 (46) | 0.23 |
| Micturition | 2 times/day | 6 (11) | 6 (11) | 0.21 |
|  | 3 times/day | 5 (9) | 11 (20) |  |
|  | 4 times/day | 15 (28) | 8 (14) |  |
|  | >4 times/day | 27 (51) | 31 (55) |  |
| **Animal Exposure/Health, and Sanitation** | | | | |
| Own a pet | Yes | 28 (52) | 40 (71) | 0.04 |
| Keep livestock | Yes | 17 (31) | 19 (34) | 0.78 |
| Observed ill livestock | Yes | 2 (4) | 3 (5) | 0.68 |
| Livestock enter bathing water | No | 22 (41) | 34 (61) | 0.05 |
|  | Yes | 13 (24) | 13 (23) |  |
|  | Unknown | 19 (35) | 9 (16) |  |
| Report pests in home | Yes | 36 (68) | 46 (84) | 0.06 |
| **Family and Past Medical History** | | | | |
| Family member CKDu (+) | Yes | 26 (49) | 32 (58) | 0.34 |
| Ever diagnosed with diabetes | Yes | 13 (24) | 2 (4) | <0.01 |
| Ever diagnosed with hypertension | Yes | 12 (22) | 29 (52) | <0.01 |
| Most villagers related | Yes | 23 (43) | 33 (59) | 0.09 |
